# Metastatic Melanoma in a Retinoblastoma Survivor: Can Immunohistochemistry Save the Day?

**DOI:** 10.7759/cureus.94424

**Published:** 2025-10-12

**Authors:** Luana-Andreea Nurla, Mariana Așchie, Cristian-Ionuț Orășanu, Mădălina Boșoteanu

**Affiliations:** 1 Dermatology, Elias Emergency University Hospital, Bucharest, ROU; 2 Pathology, Faculty of Medicine, Ovidius University of Constanta, Constanta, ROU; 3 Pathology, Sf. Apostol Andrei Emergency County Hospital, Constanta, ROU; 4 Medical Sciences, Academy of Romanian Scientists, Bucharest, ROU

**Keywords:** braf, immunohistochemistry, melanoma, p16, retinoblastoma

## Abstract

Retinoblastoma (Rb) is a rare pediatric ocular malignancy, typically diagnosed before the age of five, with unilateral or bilateral presentation. We report the complex case of a 43-year-old female patient with an oncological family history suggestive of hereditary cancer predisposition syndrome that included multiple malignancies: Rb in her great-grandmother and sister, colonic adenocarcinoma in her grandfather, and pulmonary carcinoma in her father. She was diagnosed with bilateral Rb in childhood and achromic melanoma at the age of 40, with 1.9 mm Breslow thickness, ulceration, and brisk inflammatory infiltrate, staged as pT2b. Furthermore, an axillary lymph node tumor was excised and histologically displayed malignant proliferation of small- to medium-sized cells with high mitotic activity, necrosis, vascular invasion, and neurotropism. Immunohistochemistry was required to determine histogenesis and comprised diffuse p16, SOX10, pan melanoma positivity, mild preferentially expressed antigen in melanoma (PRAME) expression, and negativity for epithelial, neuroendocrine, hematopoietic, and other lineage markers, while Ki-67 was positive in 50% of tumor nuclei. The BRAF mutation testing was negative; therefore, the final diagnosis was cutaneous achromic melanoma with lymph node metastasis, the small-cell type. Retinoblastoma survivors face increased melanoma risk, especially of second cancers. This report highlights the necessity of regular dermatological surveillance and the diagnostic utility of immunohistochemistry in identifying metastases from melanomas occurring in this patient population.

## Introduction

Retinoblastoma (Rb), initially described by Benedict, is a rare pediatric ocular malignancy originating from primitive retinal cells [1]. It typically presents within the first five years of life, with diagnoses occurring as early as in utero and extending through early childhood. Accounting for approximately 3% of all pediatric malignancies diagnosed before the age of 15, Rb demonstrates a bimodal distribution of laterality: approximately two-thirds of cases are unilateral, while the remaining third involve the ocular structures bilaterally [2]. The incidence is estimated at one in 17,000 live births, underscoring its rarity yet clinical relevance [2]. Individuals with a personal or familial history of Rb exhibit an elevated risk for developing secondary malignancies, among which cutaneous melanoma is notably prevalent, accounting for approximately 7% of second primary tumors in this population [3].

This report aims to emphasize the critical importance of systematic dermatological and dermoscopic surveillance in Rb survivors, given that early detection of potential cutaneous melanomas may significantly improve clinical outcomes. Furthermore, this article aims to underline the pivotal role of immunohistochemistry in accurately identifying metastatic lesions, particularly in patients with multiple primary melanomas (MPM), thereby facilitating precise diagnosis and guiding appropriate therapeutic strategies.

## Case presentation

Clinical findings

We report the complex case of a 43-year-old female patient, from an urban environment, with a significant oncologic background suggestive of a hereditary cancer predisposition syndrome. Her family history was notable for multiple malignancies: her great-grandmother was diagnosed with Rb, her grandfather with colonic adenocarcinoma, her father with pulmonary carcinoma, and an elder sister, who died at the age of four, was also diagnosed with Rb. The patient was also diagnosed in infancy with Rb affecting the right eye, leading to enucleation at the age of one, followed by radiotherapy after a bone relapse. At the age of nine, she developed Rb of the left eye, for which she received conservative treatment in Germany. At 30, she developed purpura of unspecified etiology. In 2021, at the age of 40, a frontal cutaneous lesion was excised and histopathologically diagnosed as achromic melanoma.

Histopathological examinations and follow-up investigations

**Figure 1:**
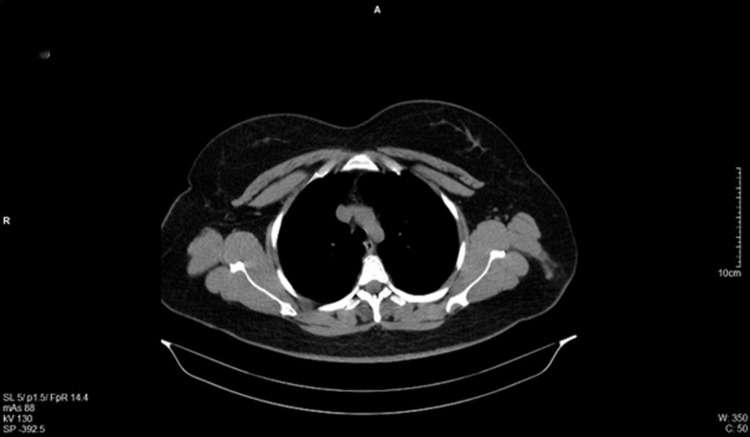
Normal axial CT scan, highlighting a section with no evidence of active disease of distant metastases (no detectable masses, lymphadenopathy, or organomegaly)

The histopathological report of the primary cutaneous melanoma revealed a Breslow thickness of 1.9 mm, ulceration, no lymphovascular or perineural invasion, a mitotic rate of 2/mm^2^, and a brisk intra- and peri-tumoral inflammatory infiltrate. There was no evidence of regression or satellitosis, and the lesion was staged as pT2b. Staging and follow-up investigations included cerebral MRI scans in 2022 and 2023, which showed no abnormalities. A cranial CT scan performed in 2022 indicated post-surgical changes in the right orbit. Thoracic, abdominal, and pelvic CT scans conducted in 2022 and 2023 showed no evidence of pathological findings (Figure 1). Laboratory investigations, including lactate dehydrogenase (LDH) and S100 in 2022, were within normal range.

A bilateral mammary ultrasound in 2023 revealed bilateral, non-vascularized fibroadenomas, classified as breast imaging-reporting and data system (BIRADS) 2. In the left breast, moderate glandular parenchyma was noted, with dilated lactiferous ducts and a well-defined, hypoechogenic, avascular oval mass measuring 1.5 × 0.3 cm, located in the retroareolar region, at the 10 o’clock position. The right breast exhibited similar glandular features, with a hypoechogenic, avascular oval mass measuring 2.3 × 0.7 cm, situated 6 cm from the mammary papilla, at the 8 o’clock position.

**Figure 2:**
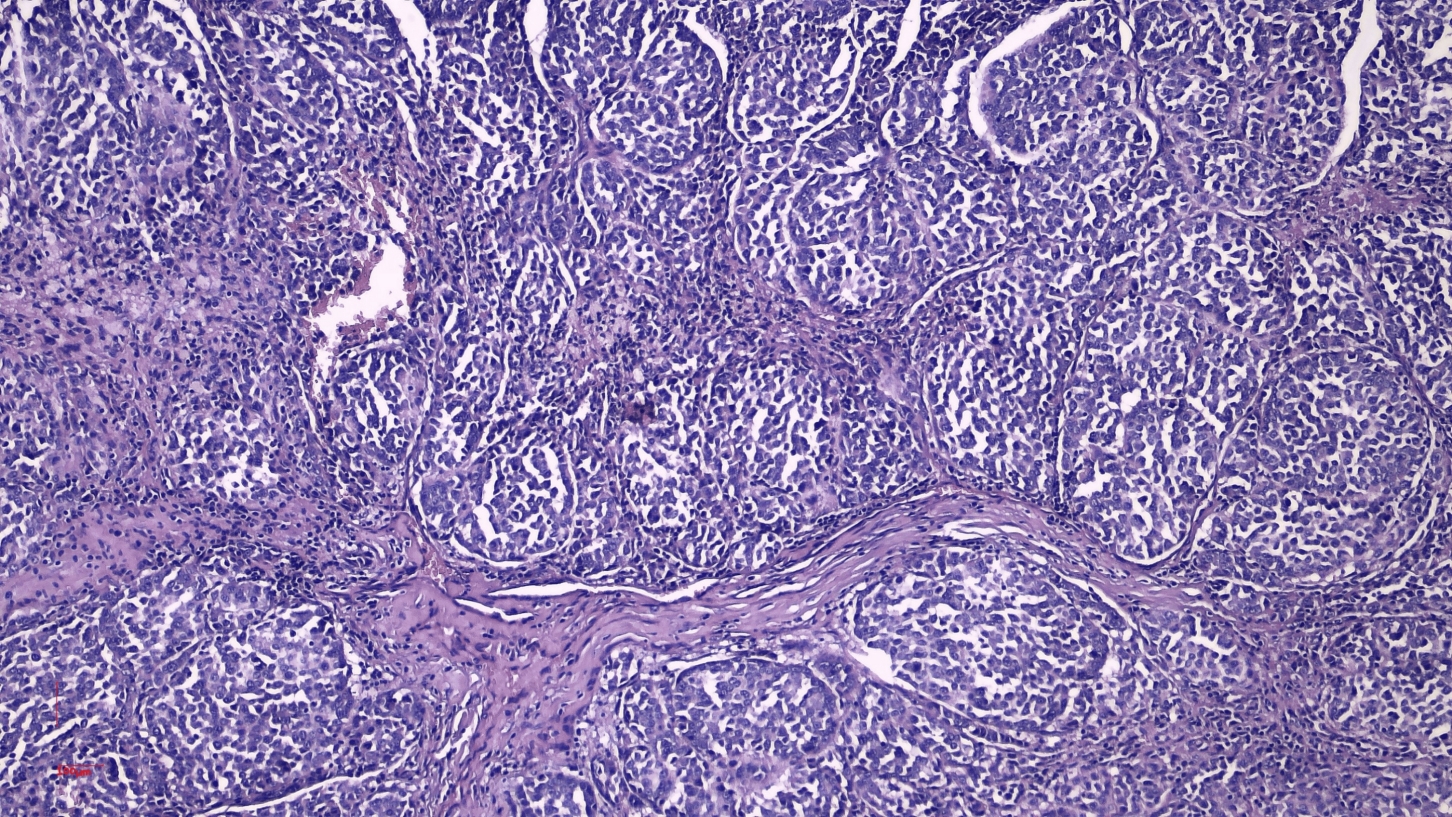
Histopathological aspect of the tumor population identified in the excised left axillary lymph node (H&E stain ×10)

In 2023, the patient underwent surgical excision of a left axillary mass. Macroscopically, the specimen was a 3.6 × 3.5 × 1.8 cm fragment of fibro-adipose tissue containing a 2.5 × 2 × 3 cm lymph node with a homogeneous whitish appearance on sectioning. The microscopic examination demonstrated a disrupted lymph node architecture due to a malignant proliferation composed of a relatively monomorphic population of small to medium-sized epithelioid cells arranged in solid, alveolar, and papillary patterns, with round-oval nuclei, inconspicuous nucleoli, and sparse eosinophilic cytoplasm. Notable features included regional discohesiveness, significant mitotic activity, extensive necrosis, a marked chronic inflammatory infiltrate, vascular invasion, and neurotropism. The maximum diameter of the tumor deposit was 20 mm. The histopathological description was initially suggestive of a lymph node metastasis from either an undifferentiated carcinoma or a high-grade nonkeratinizing squamous cell carcinoma (Figure 2). As such, additional immunohistochemical investigations were warranted to clarify the histogenesis.

A dermatological assessment conducted in 2023 found no signs of tumor recurrence, with a normal postoperative scar in the left axillary region, following the excision of the lymph node metastasis. Additional dermatological findings included angiomas, a previously cauterized papilloma, papillomatous and junctional nevi.

Immunohistochemical evaluation

**Figure 3:**
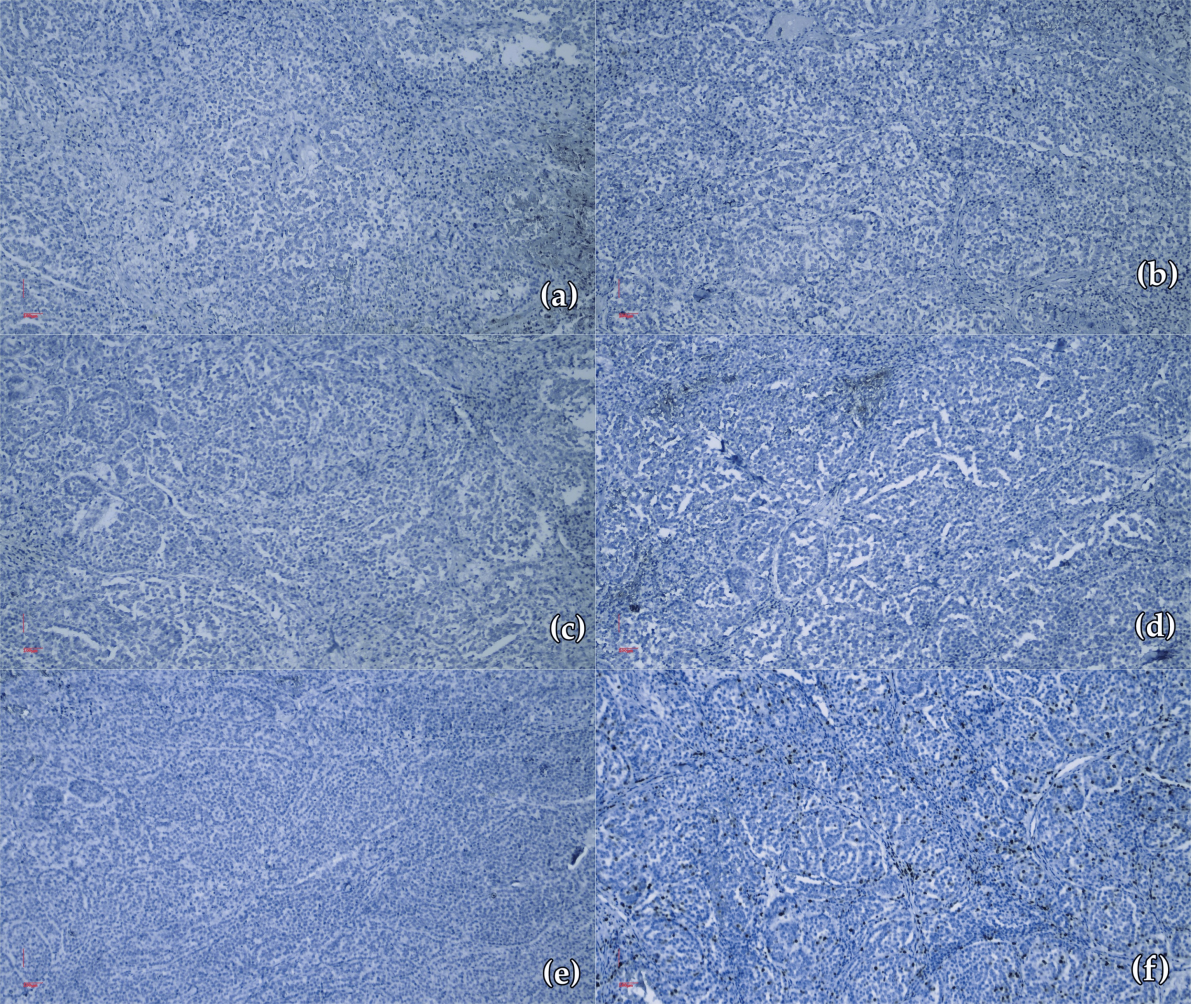
Negative immunohistochemical reactions (a) AE1/AE3 negative reaction (×10); (b) Chromogranin negative reaction (×10); (c) Synaptophysin negative reaction (×10); (d) GATA-3 negative reaction (×10); (e) TTF-1 negative reaction (×10); (f) Ki-67 positive reactions in 50% of the nuclei of the tumor population (×10) TTF-1: Thyroid transcription factor-1

A 14-marker immunohistochemical panel was applied to paraffin-embedded tissue (Table 1). The immunoprofile revealed negative AE1/AE3 (Figure 3a), chromogranin (Figure 3b), synaptophysin (Figure 3c), GATA-3 (Figure 3d), and thyroid transcription factor-1 (TTF-1) reactions (Figure 3e). The p63, CK7, CK20 also displayed negative reactions. The leukocyte common antigen (LCA) revealed a negative reaction, with positive internal control. The Ki-67 generated positive reactions in 50% of the nuclei comprised in the tumor proliferation (Figure 3f).

**Table 1:** Immunohistochemical panel used for the left axillary tissue specimen IHC: Immunohistochemical, RTU: Ready-to-use, TTF-1: Thyroid transcription factor-1, PRAME: Preferentially expressed antigen in melanoma, LCA: Leukocyte common antigen

IHC* marker	Manufacturer	Clone	Dilution	Origin	Results (tumor population)
p16	Master Diagnostica (Vitro, Sevilla, ESP)	MX007	RTU, 7 ml	Mouse, monoclonal	Diffuse expression
Pan cytokeratin	Biocare (Biocare Medical, Pacheco, CA, USA)	AE1/AE3	RTU, 6 ml	Mouse, monoclonal	Negative reaction
p63	Zeta (Zeta Corp., San Francisco, CA, USA)	ZM 70	RTU, 7 ml	Mouse, monoclonal	Negative reaction
Synaptophysin	Biocare	27G12	RTU, 6 ml	Mouse, monoclonal	Negative reaction
Chromogranin	Zeta	ZM 12	RTU, 7 ml	Mouse, monoclonal	Negative reaction
Ki-67	Zeta	MIB-1	RTU, 7 ml	Mouse, monoclonal	Nuclear positive reaction (50%)
Pan melanoma	Biocare	M2-7C10 + M2-9E3+T31	RTU, 6 ml	Mouse, monoclonal	Diffuse positive reaction
Cytokeratin 7 (CK7)	Biocare	BC1	RTU, 6 ml	Rabbit, monoclonal	Negative reaction
Cytokeratin 20 (CK20)	Biocare	Ks 20.8	RTU, 6 ml	Mouse, monoclonal	Negative reaction
LCA	Biocare	PD7/26/16 + 2B11	RTU, 6 ml	Mouse, monoclonal	Negative reaction; positive internal control
GATA-3*	Biocare	L50-823	RTU, 6 ml	Mouse, monoclonal	Negative reaction
TTF-1*	Biocare	8G7G3/1	RTU, 6 ml	Mouse, monoclonal	Negative reaction
SOX10	Biocare	BC34	RTU, 6 ml	Mouse, monoclonal	Diffusely positive reaction
PRAME*	Biocare	ZR383	RTU, 6 ml	Rabbit, monoclonal	Mildly positive reaction

**Figure 4:**
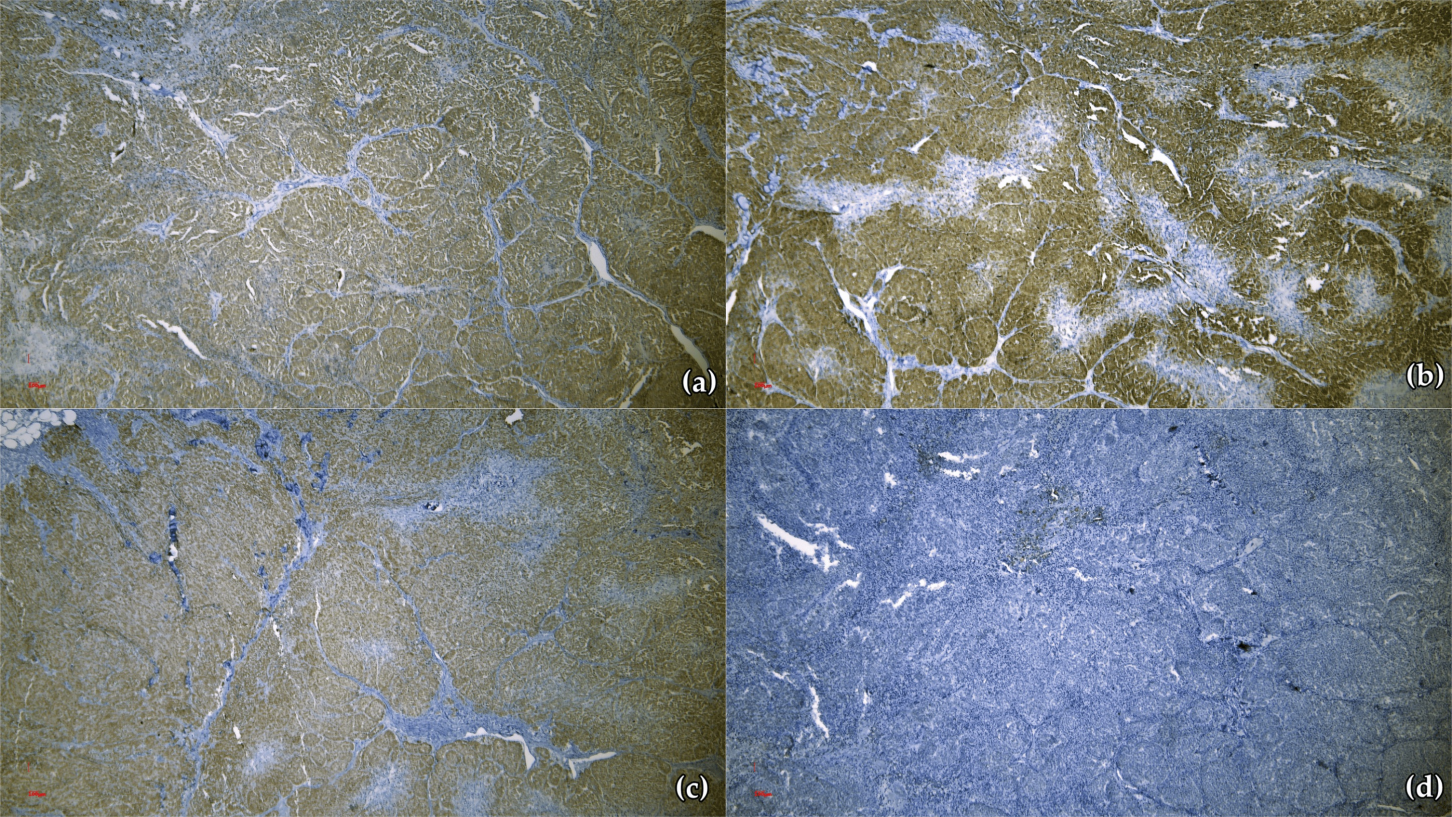
Positive immunohistochemical reactions (a) p16 diffusely present expression (×5); (b) Pan melanoma diffusely positive reaction (×5); (c) SOX10 diffusely positive reaction (×5); (d) PRAME mildly positive reaction (×10) PRAME: Preferentially expressed antigen in melanoma

Conversely, p16 exhibited diffusely present expression (Figure 4a), similar to the case of pan melanoma (Figure 4b) and SOX10 (Figure 4c), while PRAME revealed a mildly positive reaction (Figure 4d). The previously described immunoprofile confirmed the diagnosis of lymph node metastasis from an achromic melanoma, the small-cell type.

Genetic testing

The patient’s medical and family history, along with her tumor spectrum, raised suspicion for a hereditary cancer predisposition syndrome, possibly related to germline RB1 mutations or broader syndromic associations. Genetic counseling and comprehensive molecular testing were strongly recommended to further investigate the underlying genetic etiology and to guide surveillance strategies for the patient and her family. In this case, the genetic specialist recommended RB1 mutation analysis via next-generation sequencing performed on a buccal swab sample. However, this test is not currently reimbursed under the national health insurance program; consequently, the decision regarding the timing of this in-depth assessment remains at the patient’s discretion. 

Furthermore, the Li-Fraumeni syndrome (LFS), associated with germline TP53 mutations, encompasses a broad neoplastic spectrum in which melanoma has been documented; Rb does not represent a canonical or prevalent component of this condition. In the reported case, characterized by the absence of corroborating evidence for LFS, the co-occurrence of Rb and melanoma was ascribed to RB1-associated oncogenic predisposition. 

On the other hand, given its established role in melanoma pathogenesis and therapeutic implications, BRAF mutation testing was performed as part of the routine molecular evaluation in an external laboratory. The DNA was isolated from the analyzed sample using the QIAmp DNA FFPE tissue kit (QIAmp DSP DNA Mini kit; QIAGEN, Hilden, DEU). Mutation analysis of exons 11 and 15 of the BRAF gene was performed using a targeted resequencing approach (Ion AmpliSeq NQS Panel; Thermo Fisher Scientific, Waltham, MA, USA). The detection limit of the method is 2% to 5% of mutant allelic content, depending on the genomic region being analyzed. Microdissection of the tumor tissue was performed, and the material was considered suitable for analysis. The assayed sample data did not carry a mutation in exons 11 and 15 of the BRAF gene. Corroborating the clinical, histopathological, immunohistochemical, and genetic data, the final diagnosis was cutaneous achromic melanoma with lymph node metastasis.

## Discussion

The primary limitation of this article consists of the inclusion of a single patient, which does not permit broad generalization of the findings. This fact represents a direct consequence of the rarity of the Rb-melanoma association in clinical practice. Moreover, the absence of long-term follow-up data restricts conclusions regarding the prognosis. Nevertheless, the clinical relevance of this presentation renders it a noteworthy addition to the existing literature. 

Moreover, immunohistochemistry is widely accessible and may be frequently regarded as a routine technique; however, it remains an indispensable diagnostic tool that warrants deliberate application and should not be overlooked in the evaluation of such cases. A causal association between germline RB1 mutations and melanoma following Rb has been postulated for decades. Multiple cohorts of hereditary Rb survivors have shown consistently elevated melanoma incidence. Hereditary Rb patients harbor a congenital monoallelic mutation in the RB1 gene within their germline DNA, predisposing retinal cells to tumorigenesis upon acquiring a second somatic RB1 alteration [4].

In familial cases, this germline mutation is inherited, whereas in sporadic hereditary forms, it arises de novo. A small subset, comprising approximately 2% of non-hereditary Rbs, lacks RB1 mutations and exhibits MYCN amplification, indicating an alternative oncogenic pathway [5]. Notably, research by McEvoy et al. has demonstrated that chromothripsis involving chromosome 13 can disrupt the RB1 locus, serving as a potential mechanism for RB1 inactivation in cases lacking both RB1 mutations and promoter methylation [5]. Additionally, families with a strong predisposition to melanoma often carry pathogenic variants in the high-risk susceptibility genes CDKN2A and CDK4 that play pivotal roles in the cell cycle regulatory cascade governed by the RB1 pathway; this overlap suggests a biologically plausible link between RB1 dysfunction and an elevated risk for melanoma [6].

Albert et al. reported the noteworthy case of a 37-year-old male patient with a history of bilateral enucleation during early childhood due to bilateral Rb, who subsequently developed two primary cutaneous melanomas [7]. While Rb is known to cluster in families and is linked to genetic alterations on chromosome 13, increasing attention has been drawn to the elevated risk of secondary malignancies among Rb survivors, which remains a leading cause of mortality in this population [8]. These malignancies often include osteosarcoma, soft tissue sarcomas, and cutaneous melanoma [9].

A case previously reported by Belt et al. describing the coexistence of Rb, dysplastic nevus syndrome (DNS), and multiple cutaneous melanomas underscores the potential interplay between these conditions and highlights a markedly increased risk for melanoma development [10]. The convergence of inherited Rb and DNS appears to constitute a significant predisposing factor for cutaneous melanoma, with implications that extend to family members. These observations support the recommendations for vigilant dermatological surveillance of Rb survivors, as well as their first-degree relatives, to enable early detection and timely intervention.

Patients with hereditary Rb exhibit a markedly heightened and earlier-onset susceptibility to both cutaneous melanoma and non-melanoma skin cancer (NMSC), with median ages at diagnosis approximately two decades younger than those observed in nonhereditary counterparts [11]. Melanomas in this population demonstrate a widespread anatomical distribution, akin to that seen in familial melanoma syndromes, while NMSCs predominantly involve the head and neck region. The occurrence of MPMs and their anatomical distribution among heritable Rb survivors mirrors patterns observed in melanoma-prone families carrying CDKN2A or CDK4 mutations, further supporting potential shared genetic susceptibilities [12]. At five decades post-diagnosis, cumulative incidence rates among hereditary survivors reach 4.5% for melanoma and 3.7% for NMSC, substantially exceeding the 0.7% and 1.5%, respectively, observed in nonhereditary individuals [13]. These findings highlight the probable contribution of an underlying genetic predisposition, independent of sun sensitivity or phenotypic features, in the pathogenesis of skin malignancies in this high-risk group. In addition, the distinction between undifferentiated carcinoma, high-grade nonkeratinizing squamous cell carcinoma (SCC), and metastatic melanoma represents a diagnostic challenge, particularly in small biopsy specimens or metastatic sites lacking obvious primary lesions. Accurate differentiation is critical due to the divergent clinical behavior, therapeutic strategies, and prognosis associated with each entity.

As a rare and distinct histopathological entity with a characteristic immunohistochemical profile, SMARCA4-deficient undifferentiated neoplasm (SD-UMN) usually consists of a highly cellular dermal neoplasm composed of pleomorphic epithelioid cells with prominent mitotic activity and necrosis, lacking clear morphological differentiation [13]. In a case reported by Gumusgoz et al. [13], there was a complete loss of SMARCA4 and SMARCA2 expression, with preserved integrase interactor 1 (INI-1). Additional findings included diffuse p53 expression and complete loss of p16. All other lineage-specific markers tested, including cytokeratins, p63, SOX10, neuroendocrine, melanocytic, myogenic, and hematolymphoid antigens, were negative.

From a histopathological perspective, high-grade nonkeratinizing SCC displays sheets or nests of pleomorphic basaloid and polygonal cells with high nuclear-to-cytoplasmic ratios and brisk mitotic activity but lacks overt keratinization [14]. These types of tumors typically express broad-spectrum cytokeratins such as AE1/AE3, as well as high molecular weight cytokeratins and nuclear markers, such as p63 and p40 [15]. Among these, p40 is more specific for squamous differentiation and is particularly useful in excluding non-squamous tumors [16].

In contrast, metastatic melanoma may show either epithelioid or spindle-cell morphology with prominent nucleoli, and melanin pigment may be observed in a subset of cases [17]. Amelanotic variants, however, may mimic undifferentiated or squamous carcinomas, further complicating the diagnostic process. Metastatic melanoma is characteristically negative for cytokeratins and squamous markers but shows strong positivity for melanocytic antigens, including S100, SOX10, Melan-A, HMB-45, and MITF [18]. Among these, SOX10 and S100 have emerged as particularly sensitive and specific parameters for melanocytic differentiation, including amelanotic tumors [19]. When both epithelial and melanocytic markers are absent, other differential diagnoses, such as lymphoma, sarcoma, or Merkel cell carcinoma, should be considered, necessitating broader immunohistochemical panels. 

The current guidelines strongly recommend complex surveillance protocols for individuals with heritable Rb, including annual physical examinations with a thorough skin assessment alongside education regarding the risks and early signs and symptoms of potential associated malignancies, particularly melanomas during childhood and adolescence. Given the minimal risk associated with total body skin examinations, and considering the higher prevalence of visual impairment in this population that may alter their capacity for monthly cutaneous auto-examination, annual dermatological visits are advised. Moreover, healthcare providers should offer reproductive genetic counseling and initiate early tumor surveillance for at-risk offspring, beginning in the prenatal period and continuing through the immediate postnatal phase.

## Conclusions

The presented case highlights a potentially underrecognized association between Rb and cutaneous melanoma, thereby warranting further investigation into underlying shared genetic susceptibilities and the development of targeted surveillance protocols for this patient population. A comprehensive approach combining histological features, a focused immunohistochemical panel, and clinical context is essential in distinguishing metastatic melanoma from undifferentiated carcinoma and high-grade squamous cell carcinoma, due to the fact that misclassification may lead to significant therapeutic consequences, particularly with the emergence of targeted treatments and immunotherapies for melanoma.
